# Despite increasing aldosterone, elevated potassium is not necessary for activating aldosterone‐sensitive HSD2 neurons or sodium appetite

**DOI:** 10.14814/phy2.14714

**Published:** 2021-01-19

**Authors:** Frederico S. Fazan, Eduardo Colombari, Arthur D. Loewy, Joel C. Geerling

**Affiliations:** ^1^ Department of Neurology Iowa Neuroscience Institute University of Iowa Hospital and Clinics Iowa City Iowa USA; ^2^ Department of Physiology and Pathology São Paulo State University Araraquara Brazil; ^3^ Department of Neuroscience Washington University School of Medicine in Saint Louis St Louis Missouri USA

**Keywords:** aldosterone, dietary sodium, mineralocorticoid, potassium, salt appetite, salt hunger

## Abstract

Restricting dietary sodium promotes sodium appetite in rats. Prolonged sodium restriction increases plasma potassium (pK), and elevated pK is largely responsible for a concurrent increase in aldosterone, which helps promote sodium appetite. In addition to increasing aldosterone, we hypothesized that elevated potassium directly influences the brain to promote sodium appetite. To test this, we restricted dietary potassium in sodium‐deprived rats. Potassium restriction reduced pK and blunted the increase in aldosterone caused by sodium deprivation, but did not prevent sodium appetite or the activation of aldosterone‐sensitive HSD2 neurons. Conversely, supplementing potassium in sodium‐deprived rats increased pK and aldosterone, but did not increase sodium appetite or the activation of HSD2 neurons relative to potassium restriction. Supplementing potassium without sodium deprivation did not significantly increase aldosterone and HSD2 neuronal activation and only modestly increased saline intake. Overall, restricting dietary sodium activated the HSD2 neurons and promoted sodium appetite across a wide range of pK and aldosterone, and saline consumption inactivated the HSD2 neurons despite persistent hyperaldosteronism. In conclusion, elevated potassium is important for increasing aldosterone, but it is neither necessary nor sufficient for activating HSD2 neurons and increasing sodium appetite.

## Graphical abstract

1. Dietary sodium deprivation activates aldosterone‐sensitive neurons and causes sodium appetite.
2. Potassium greatly influences aldosterone levels in sodium‐deprived rats.
3. The activation of aldosterone‐sensitive neurons does not correlate with plasma potassium.
4. Sodium appetite does not require increased plasma potassium.
5. In sodium‐deprived rats, increasing or reducing plasma potassium and aldosterone does not significantly alter sodium appetite or the activation of aldosterone‐sensitive neurons.

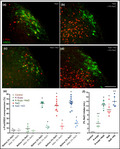

## 1 INTRODUCTION

Sodium appetite is a complex, motivated behavioral state. It requires low‐sodium status detection and a coordinated drive to seek and ingest salty foods. Interestingly, this elaborate behavior can be elicited by administering a singular hormone, aldosterone (Formenti et al., 2013; Gasparini et al., 2018; Geerling & Loewy, 2008). Aldosterone is a mineralocorticoid produced in the zona glomerulosa of the adrenal cortex. It affects cells that express the mineralocorticoid receptor (MR) by translocating this receptor into the cell nucleus to promote genomic activity (Robertson et al., 1993). Aldosterone‐sensitive cells also require the enzyme 11‐beta‐hydroxysteroid dehydrogenase type 2 (HSD2), which protects the MR from glucocorticoids, leaving it more accessible to aldosterone (Funder et al., 1988; Naray‐Fejes‐Toth et al., 1998; Odermatt et al., 2001). Therefore, HSD2 is a biological marker for aldosterone‐sensitive cells.

A small number of neurons in the brain express both MR and HSD2. These cells, referred to as HSD2 neurons and located in the nucleus of the solitary tract, drive sodium appetite (Geerling et al., 2006; Jarvie & Palmiter, 2017; Resch et al., 2017). Infusing aldosterone activates them and elicits sodium appetite in rats (Formenti et al., 2013; Gasparini et al., 2018), and destroying them reduces sodium appetite in mice (Resch et al., 2017). Importantly, however, their activation and sodium appetite still occur in adrenalectomized rats with no detectable aldosterone (Geerling, Engeland, et al., 2006; Jalowiec & Stricker, 1973; Rice & Richter, 1943). The primary signal that activates HSD2 neurons remains unknown.

Sodium itself would seem a logical signal for sodium deficiency, but dietary sodium restriction typically does not decrease the concentration of sodium in the blood plasma (Contreras & Hatton, 1975; Francesconi & Hubbard, 1985; Stricker et al., 1991). This is due to a proportional contraction of the overall volume of extracellular fluid (ECF), which prevents the sodium concentration from decreasing (Francesconi & Hubbard, 1985; Gupta et al., 1981). Also, decreasing the ECF sodium concentration does not increase sodium appetite (Stricker & Wolf, 1966), nor does it augment the activation of HSD2 neurons in sodium‐deprived rats (Geerling & Loewy, 2007a), indicating that sodium itself is not the key signal promoting sodium appetite.

In contrast, potassium rises in the blood plasma after prolonged sodium restriction (Contreras & Hatton, 1975; Francesconi & Hubbard, 1985), but the possibility that this rise in potassium promotes sodium appetite remains unexplored. Potassium, along with angiotensin II, is a critical signal for aldosterone secretion (Okubo et al., 1997; Yang et al., 2016). Elevated ECF potassium promotes membrane depolarization in the adrenal zona glomerulosa, triggering voltage‐dependent mechanisms that ultimately induce aldosterone release (Lotshaw, 2001; Yang et al., 2016, 2018). Aldosterone secretion requires a change in potassium current across the cell membrane (Yang et al., 2018), increasing roughly 100‐fold per 1 mM rise in extracellular potassium (Lotshaw, 2001). In human patients, the importance of potassium for aldosterone release was solidified by the discovery that a frequent cause of primary hyperaldosteronism is a somatic *KCNJ5* mutation that disrupts the potassium‐selectivity of the Kir3.4 channel, allowing sodium entry through this channel to inappropriately depolarize aldosterone‐secreting cells (Choi et al., 2011). In rodents, the importance of potassium was clear from the observation that hyperaldosteronism caused by dietary sodium restriction is nearly blocked when the elevation in ECF potassium is prevented by simultaneous dietary potassium restriction (Okubo et al., 1997).

All excitable cells are vulnerable to increases in the ECF concentration of potassium. In neurons, membrane depolarization caused by elevated ECF potassium typically increases spiking activity (Macias et al., 2001; Rutecki et al., 1985). Fortunately, regulatory mechanisms usually prevent this by maintaining the ECF concentration of potassium within a strict physiological range. As an added line of defense, the blood–brain barrier (BBB) protects neurons from peripheral potassium fluctuations. However, the HSD2 neurons are in a BBB‐deficient region, where the extracellular environment more closely resembles blood plasma (Broadwell & Sofroniew, 1993; Gasparini et al., 2019; Gross et al., 1990). Besides increasing their access to circulating aldosterone, this BBB‐deficient location may expose the HSD2 neurons to shifts in circulating potassium, which could serve as a useful signal for sodium deprivation. Our study aimed to investigate whether a rise in circulating potassium during dietary sodium restriction promotes HSD2 neuronal activation and sodium appetite.

In sodium‐deprived rats, we manipulated dietary potassium to test the hypothesis that elevated potassium not only boosts aldosterone but also is *necessary* for activating the HSD2 neurons and increasing sodium appetite. We placed rats on a simultaneously low‐sodium and low‐potassium diet, which prevented plasma potassium from rising during sodium deprivation. Also, to test whether elevated potassium is *sufficient* to increase HSD2 neuron activity and sodium appetite, we supplemented dietary potassium, both with and without simultaneous dietary sodium deprivation. We measured c‐Fos activation of HSD2 neurons and assessed sodium appetite along with other trends in fluid intake, body weight, plasma electrolytes, total protein, and plasma aldosterone. Despite its substantial effects on aldosterone, elevated potassium was neither necessary nor sufficient for HSD2 neuronal activation or sodium appetite in sodium‐deprived rats.

## 2 MATERIALS AND METHODS

### 2.1 Animals

Animal husbandry and all experimental protocols were approved by the Washington University School of Medicine Institutional Animal Care and Use Committee (protocol #20080299) and conformed to NIH guidelines. All experiments involved young‐adult, male Sprague–Dawley rats (225–250 g; Harlan). Rats were group‐housed for at least 3 days upon arrival. During this time, they had ad libitum access to tap water and standard rat chow (PicoLab rodent diet 20; LabDiet). Rats were used in just one experiment each, such that every experimental group represents a new case with no prior dietary manipulations. At the end of each experiment, every rat was perfused for brainstem histology as described below. Rats were housed at room temperature on an automated 12/12‐h light/dark cycle, with lights on and off at 6:30 a.m. and 6:30 p.m., respectively.

### 2.2 Dietary experiments

Prior to each experiment, rats were separated into individual cages for a baseline period of up to 4 days. During this time, standard chow was available ad libitum, in addition to two graduated drinking tubes. One tube contained distilled water (dH_2_O), and the other tube contained concentrated saline (3% NaCl, ~0.5 M). The fluid meniscus was recorded daily for 10 days, beginning 2 days prior to the 8‐day experimental period and continuing through the morning of perfusion.

Initially, all rats had ad libitum access to a standard rodent chow, which contains 1.1% potassium and 0.3% sodium (PicoLab 20, #5053, LabDiet). For potassium‐supplemented groups, we left this chow in place until the end of the experiment. To simultaneously restrict potassium and sodium, we used a custom diet containing 0.003%–0.008% K and 0.010%–0.015% Na (TD.86304, Harland‐Teklad). To restrict only sodium, we used a custom modification of this diet with added potassium (10 g/kg K‐citrate; TD.06102, Harlan‐Teklad). To supplement dietary potassium, we replaced the drinking water with potassium chloride (3% KCl), after pilot tests in *n* = 4 rats maintained on low‐sodium chow confirmed that they maintain normal appearance and behavior while consuming either 2% or 3% KCl as the sole source of liquid.

**FIGURE 1:**
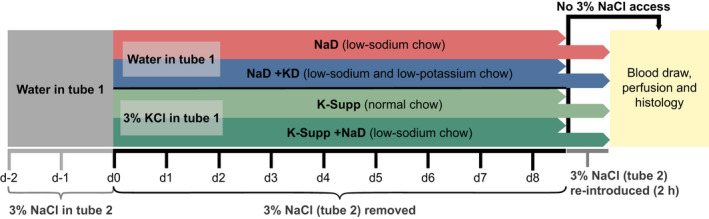
Experimental protocol. Primary endpoints were (a) activation of HSD2 neurons, estimated using nuclear immunoreactivity for c‐Fos, and (b) sodium appetite, estimated from 3% NaCl consumption in rats given 2 h access to saline. Figure S2 shows the experimental protocol for a subsequent pair of comparison groups given uninterrupted access to 3% NaCl

As summarized in Figure 1, to begin the 8‐day experimental period of dietary deprivation or supplementation, we removed the 3% NaCl tube from each cage, while continuing ad libitum access to both chow and dH_2_O (or 3% KCl, in potassium‐supplemented groups). Bodyweight and fluid intake volumes were measured every morning, between 8–10 a.m., beginning 48 h prior to removal of saline, so as to include a 2‐day baseline. Clean cages were provided daily to minimize the ingestion of excreted sodium or potassium. Between 6 and 7 a.m. at the end of the 8th day, we drew blood and transcardially perfused a subset of rats (every other cage), as described below. In the remaining cages, we began a 2 h sodium‐appetite test by replacing the saline drinking tube (clean, with fresh 3% NaCl) that had been removed 8 days prior. We recorded the fluid meniscus on the water tube and the freshly replaced saline tube at that time, then +30 min, +1 h, and +2 h. At the end of this 2 h sodium appetite test, we drew blood from and transcardially perfused these rats, as described below. Although we were able to draw blood and perfuse rats rapidly and continuously in two parallel setups, the time for anesthesia and perfusion required that the total time from saline reintroduction to perfusion ranged up to 2.5 h in some rats tested for sodium appetite. Additional control groups were treated similarly, except that the 3% NaCl drinking tube remained continuously available until the morning of perfusion, with dH_2_O and 3% NaCl consumption recorded daily throughout the experiment.

All perfusions and drinking tests were initiated early in the morning (shortly after 6:30 a.m., which was the end of the 12 h dark period, when rats are primarily awake) because later in the day the rats are typically sleeping, which results in more variable and overall less drinking behavior and less c‐Fos activation in most brain regions (Chae & Heideman, 1998; Geerling & Loewy, 2007b).

### 2.3 Blood samples, perfusions, and histology

At the end of every experiment, each rat was anesthetized with pentobarbital (50 mg/kg, i.p.). To prevent clotting, we first infused heparin (50 U in 0.5 ml; Abbot) via a femoral catheter, then a blood sample (~3 ml) was drawn directly from the left ventricle of the heart immediately prior to perfusion. Blood plasma was immediately separated by centrifugation at 4°C (5000 rpm for 5 min). The plasma was used for the determination of protein, sodium, potassium, and aldosterone concentrations, all performed in the clinical laboratories at Washington University (Barnes‐Jewish Hospital). Immediately after the blood draw, each rat was perfused, through a blunt cannula inserted into the ascending aorta, with isotonic saline followed by 4% paraformaldehyde in 0.1 M sodium phosphate buffer (pH 7.4). Brains were postfixed in 4% paraformaldehyde overnight, sectioned the following day on a freezing microtome, and then loaded into primary antisera to label HSD2 and c‐Fos.

A 1‐in‐5 series of sections, cut in the axial (transverse) plane and extending from 500 µm rostral to the area postrema back through the spinomedullary transition, containing the full extent of HSD2 neurons in the NTS, was used for double‐immunofluorescence staining. To label the HSD2 neurons, we used an affinity‐purified sheep polyclonal antiserum raised against a synthetic peptide from rat 11‐beta‐hydroxysteroid dehydrogenase type 2 (1:40,000, #1296 Chemicon). This antiserum was raised against a recombinant protein generated from nucleotides 385–1204 of the rat HSD2 gene (see Gomez‐Sanchez et al., 2001). At a 1:40,000 dilution, it produces optimal signal‐to‐noise labeling of the restricted group of HSD2‐expressing neurons in the NTS, in agreement with the pattern of *Hsd11b2* mRNA in rats (Geerling et al., 2006, 2010; Roland et al., 1995). We combined this with a rabbit antibody to label c‐Fos (1:10,000, “Ab‐5” from Oncogene; later renamed “PC38” by Calbiochem and no longer commercially available). This antiserum was raised against a synthetic peptide (SGFNADYEASSSRC) corresponding to amino acids 4–17 of human c‐Fos. We combined these two primary antisera in a solution of phosphate‐buffered saline (PBS) containing Triton‐X100, 5% normal donkey serum (Jackson Immunoresearch), and 0.1% sodium azide (Sigma). Each section was incubated in a separate well of a 9‐well glass plate containing 100 µl of primary antibody solution. After overnight incubation on a shaker at room temperature, sections were washed twice in PBS and incubated for an additional 2 h in a secondary antibody solution containing Cy2‐donkey‐anti‐sheep and Cy3‐donkey‐anti‐rabbit (each diluted 1:500; Jackson ImmunoResearch) in the same base solution as above (PBS with Triton‐X100, donkey serum, and azide). After two additional PBS washes, sections were mounted on glass slides and coverslipped with glycerol‐based mounting media, then imaged and analyzed the same day.

### 2.4 Imaging, figures, and data analysis

After immunohistochemical staining, slides were reviewed and plotted in a Nikon epifluorescence microscope at 400× magnification. All HSD2 neurons containing a nucleus within the plane of section were counted and scored for the presence or absence of nuclear c‐Fos‐immunoreactivity using a digital X‐Y plotter system (Accustage). The number of activated HSD2 neurons (those containing a c‐Fos‐ir nucleus) was divided by the total number of HSD2 neurons containing a nucleus within the plane of section, resulting in a percent‐estimate of HSD2 neuronal activation in that animal (Geerling & Loewy, 2007a, 2007b).

Data are presented as group mean ± standard deviation. Each preplanned, individual statistical comparison between two means was performed using Student's two‐tailed *t*‐test with type I error probability of *p* < 0.05 considered statistically significant. Comparisons across more than two groups and all secondary or unplanned, *post hoc* comparisons were performed using ANOVA followed by Tukey's correction for multiple comparisons. In a small number of cases, hemolysis necessitated excluding the blood sample from analysis due to grossly elevated plasma potassium values that were several standard deviations above the group mean. Also, for several rats, insufficient plasma prevented our clinical laboratory from running a repeat, diluted sample for plasma aldosterone after the initial, undiluted result surpassed the upper limit of the standard curve; we excluded these aldosterone values (“>70 ng/dl”) from further analysis.

Brightfield images were taken using a Nikon epifluorescence microscope equipped with a CCD camera and Nikon ACT‐1 software (v2.62). We used Adobe Photoshop to combine and pseudocolor grayscale, single‐channel fluorescence images and to perform uniform brightness and contrast adjustments. Data tables from Microsoft Excel were exported to GraphPad Prism to produce graphs and perform statistical analyses. We used Adobe Illustrator for figure layouts.

## 3 RESULTS

**FIGURE 2:**
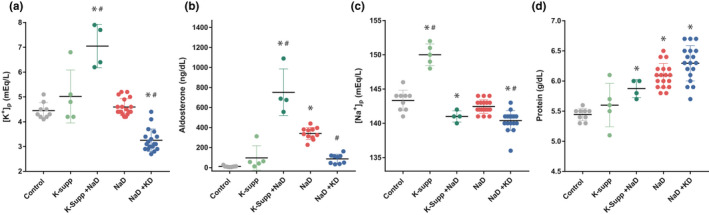
Blood plasma values of (a) potassium, (b) aldosterone, (c) sodium, and (d) total protein. Asterisks indicate a significant difference from controls. Hash symbols indicate a significant difference from the sodium‐deprived (NaD) group

**FIGURE 3:**
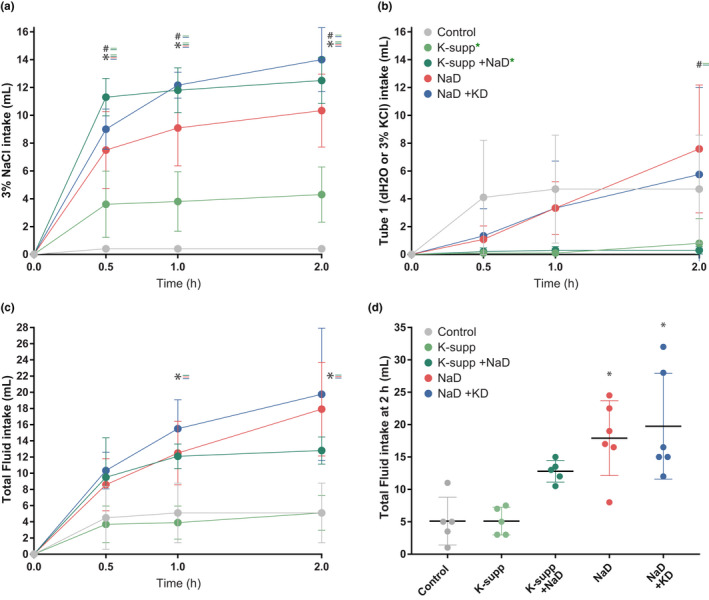
Effects of dietary manipulations on sodium appetite. Consumption of (a) concentrated saline (3% NaCl) and (b) water (or 3% KCl, in potassium‐supplemented groups, indicated by green asterisks) over a 2 h sodium appetite test. Potassium supplementation slightly elevated 3% NaCl intake, but potassium deprivation did not reduce 3% NaCl intake in sodium‐deprived rats. (c, d) Total fluid volumes for each group. Asterisks indicate a significant difference from controls. Hash symbols indicate a significant difference from the sodium‐deprived (NaD) group

**FIGURE 4:**
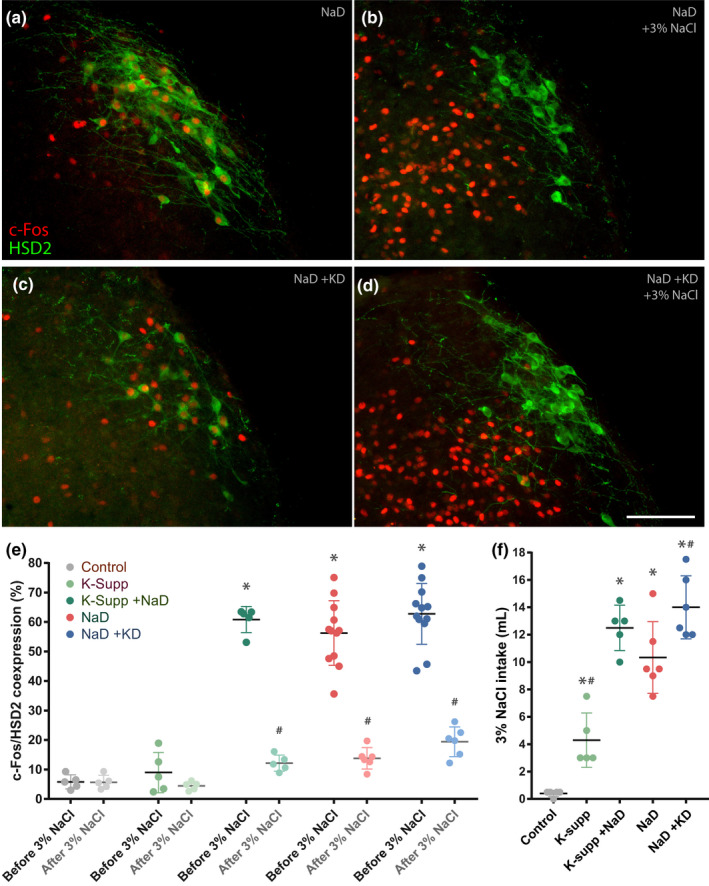
Activation of aldosterone‐sensitive HSD2 neurons. (a, c) Dietary sodium deprivation increases c‐Fos (red) nuclear immunoreactivity in neurons in the nucleus of the solitary tract (NTS), which is immunoreactive for 11‐beta‐hydroxysteroid dehydrogenase type 2 (HSD2, green). (b, d) Saline ingestion greatly increases c‐Fos nuclear immunoreactivity in the medial NTS, while reducing c‐Fos in HSD2 neurons. Examples in the top row are from sodium‐deprived rats (NaD), with (a) or without (b) 2‐h access to a saline drinking tube. Examples in the second row are from sodium‐ and potassium‐deprived rats (NaD+KD), with (a) or without (b) 2 h access to saline. There were no qualitative differences in c‐Fos activation produced by sodium deprivation with or without concurrent potassium deprivation. (e) The large increase in HSD2 neuronal activation produced by NaD, as measured by nuclear immunoreactivity for c‐Fos, was not significantly different in groups that were simultaneously potassium‐supplemented (NaD+K‐supp) or potassium‐deprived (NaD+KD). In all NaD groups, 3% NaCl consumption resulted in a significant decrease in HSD2 neuronal activity. (f) Shown, for reference, are the total intake volumes of 3% NaCl of the subgroups given a 2 h sodium appetite test before perfusion. Asterisks indicate a significant difference from controls. Hash symbols in panel (e) indicate a significant difference from the same group before 3% NaCl. Hash symbols in panel (f) indicate a significant difference from the sodium‐deprived (NaD) group

Altering dietary sodium and potassium evoked complex changes in blood chemistry, neuronal activation, and ingestive behavior. In addition to fluid intake and body weight during the experimental period (Figure S1), we analyzed plasma levels of aldosterone, potassium, sodium, and total protein (Table 1 and Figure 4), along with our primary endpoints, sodium appetite (Figure 2), and HSD2 neuronal activation (Figures 3, 4).

**TABLE 1:** Mean values for potassium (pK), aldosterone (Aldo), sodium (pNa), and total protein in blood plasma from rats in each group.

	pK (mM)	Aldo (ng/dl)	pNa (mM)	Protein (g/dl)
Control (*n* = 9)	4.4 ± 0.3	11 ± 7	143 ± 2	5.4 ± 0.1
K‐supp (*n* = 5)	5.0 ± 1.0	97 ± 122	150 ± 2^a^, ^b^	5.6 ± 0.4^b^
NaD+K‐supp (*n* = 4)	7.1 ± 0.9^a^	753 ± 233^a^, ^b^	141 ± 1^a^	5.9 ± 0.2
NaD (*n* = 19 total)	4.6 ± 0.3	341 ± 60^a^ (*n* = 11)	142 ± 1	6.1 ± 0.2^a^
NaD+KD (*n* = 18 total)	3.3 ± 0.4^a^	87 ± 46^b^ (*n* = 9)	140 ± 1^a^	6.3 ± 0.3^a^

Across the 8 days experimental period, control rats had regular chow and water, K‐supp (potassium‐supplemented) had regular chow and 3% KCl, NaD+K‐supp (sodium deprived, potassium‐supplemented) had low‐sodium chow and 3% KCl, NaD (sodium‐deprived) had low‐sodium chow and water, and NaD+KD (sodium‐deprived and potassium‐deprived) had low‐sodium and low‐potassium chow and water. Group numbers are indicated for aldosterone in two groups (NaD and NaD+KD) for which this hormone was not measured in all cases. Each experimental group was compared to the control group and NaD group using a one‐way ANOVA followed by Tukey's correction for multiple comparisons.

^a^ Versus control.

^b^ Versus NaD.

### 3.1 Blood plasma

Dietary sodium restriction increased the plasma protein concentration by approximately 12% (Table 1; Figure 2D), evidencing a reduction in ECF volume. This effect was not altered by supplementing or restricting potassium.

Dietary sodium restriction alone did not reduce the plasma sodium concentration (pNa), which fell slightly after simultaneously supplementing or restricting dietary potassium (Table 1; Figure 2C). Potassium supplementation without sodium restriction greatly increased pNa, without significant changes in aldosterone, pK, or total protein.

As expected, restricting dietary sodium produced a large increase in plasma aldosterone, averaging 30‐fold above control values (Table 1; Figure 2B). In sodium‐restricted rats that were supplemented with potassium, this increase in aldosterone was even larger, roughly double the sodium‐deprived group and almost 70‐fold higher than controls. Conversely, restricting dietary potassium blunted the increase substantially.

Effects on plasma potassium (pK) were similarly large. Potassium supplementation alone did not produce a consistent increase, but combining potassium supplementation with sodium deprivation greatly elevated pK (Table 1; Figure 2A). Conversely, restricting potassium in sodium‐deprived rats reduced pK well below control values. Dietary sodium restriction for 8 days did not significantly elevate pK, in contrast to more extended time periods (Francesconi & Hubbard, 1985).

### 3.2 Sodium appetite

Restricting dietary sodium increased sodium appetite (Figure 3A). Restricting potassium did not reduce sodium appetite. In fact, saline intake was slightly higher in rats that were simultaneously deprived of potassium and sodium, despite their reduced pK and blunted aldosterone. Potassium supplementation in sodium‐deprived rats evoked slightly more saline intake than sodium deprivation alone, but no more than sodium deprivation combined with potassium restriction. Supplementing potassium without restricting sodium increased saline intake slightly (Figure 3A) without significantly increasing total fluid intake during the 2 h test (Figure 3C,D).

### 3.3 Activation of HSD2 neurons

Restricting dietary sodium increased the proportion of HSD2 neurons containing nuclear immunoreactivity for c‐Fos in every group (Figure 4A). With or without simultaneous alterations in dietary potassium, HSD2 neuronal activation was similar (Figure 4E). Lowering pK by restricting dietary potassium (Figure 4C) did not reduce HSD2 neuronal activation, nor did raising pK by supplementing potassium increase it beyond what appeared to be a ceiling of roughly 60%–80%. Supplementing potassium without sodium deprivation did not increase c‐Fos activation of HSD2 neurons significantly above control levels. Each sodium‐deprived subgroup that was allowed to consume saline (Figure 4F) had a large reduction in the percentage of HSD2 neurons with nuclear c‐Fos immunoreactivity (Figure 4E), contrasting a broad increase in c‐Fos immunoreactivity in many nearby NTS neurons population (Figure 4B,D).

### 3.4 Potassium, aldosterone, and HSD2 neuronal activation

**FIGURE 5:**
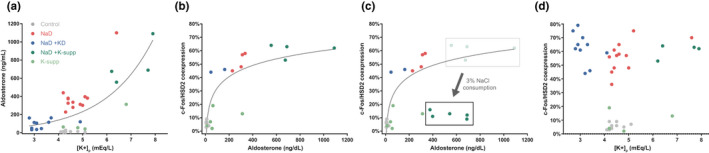
(a) Relationship between plasma values of potassium and aldosterone across groups. Despite an apparently positive association with aldosterone (a), plasma potassium had no apparent correlation with c‐Fos activation of the HSD2 neurons in that HSD2 neuronal activation was similarly elevated in rats that were deprived of dietary sodium across a wide range of potassium values (d). (b) Relationship of plasma aldosterone and c‐Fos activation of HSD2 neurons across groups. (c) In one group (K‐supp +NaD), we measured plasma aldosterone both with and without 2 h access to 3% NaCl, and found that HSD2 neuronal activation reduced sharply despite persistently elevated plasma aldosterone

A scatterplot relating plasma values across groups revealed what appeared to be a positive relationship between pK and aldosterone (Figure 5A). In contrast, there was no apparent correlation between pK and c‐Fos activation of HSD2 neurons (Figure 5D). Aldosterone and HSD2 neuronal activation appeared somewhat correlated, but the strength of this relationship diminished above aldosterone levels of approximately 300 ng/dl (Figure 5B). Of note, drinking saline reduced c‐Fos activation of HSD2 neurons, even in rats with the highest aldosterone values in our study (Figure 5C).

### 3.5 Potassium deprivation alone

In two additional groups, we fed rats a low‐sodium chow (*n* = 4) or chow that lacks both sodium and potassium (*n* = 4), but we left the 3% NaCl tube in all cages at all times. Because both groups had continuous saline access, rats with chow lacking sodium and potassium were deprived of only potassium, and rats with low‐sodium chow served as nondeprived controls. Potassium deprivation reduced pK (2.5 ± 0.3 mM vs. 4.4 ± 0.3 mM, *p* < 0.001 by two‐tailed *t*‐test), and slightly increased the plasma protein concentration (5.8 ± 0.2 mM vs. 5.4 ± 0.1 mM, *p* < 0.001) without changing pNa, aldosterone, or c‐Fos in HSD2 neurons. Potassium‐deprived rats drank an increased volume of 3% NaCl every day (averaging 8.1 ± 1.1 ml per 24 h; Figure S2), similar to previous reports (Adam & Dawborn, 1972; Blake & Jurf, 1968; Zucker, 1965). Control rats with low‐sodium chow drank less 3% NaCl (averaging 2.5 ± 0.5 ml per 24 h; *p* < 0.001).

## 4 DISCUSSION

Uncertainty regarding the signals linking sodium deprivation to HSD2 neuronal activation and sodium appetite motivated this study. HSD2 neurons do not require aldosterone, and conditions that promote sodium appetite typically do not involve a change in the concentration of sodium. Conversely, an increase in pK caused by prolonged sodium deficiency is the primary stimulus for aldosterone release. Our results confirm the importance of potassium for increasing aldosterone, and we confirm that dietary sodium restriction activates HSD2 neurons and promotes sodium appetite. However, as discussed below, our findings do not support potassium as an important signal for HSD2 neurons or sodium appetite.

### 4.1 Limitations

As much as possible, we tested experimental rats and matched controls side‐by‐side, in full cohorts, with samples collected and processed in parallel. However, the practical constraints of obtaining sufficient blood plasma for analysis, plus drinking behavior and high‐quality histology, required that we run repeat cohorts for some experiments. The number of rats with a full suite of plasma measurements was limited in some groups, particularly for aldosterone, reducing our statistical power to detect small changes in secondary endpoints. Our primary goal in this study was not to characterize small effects, but rather to learn whether sodium appetite or HSD2 neuronal activation require elevated potassium, and our results clearly show that potassium is not a critical signal for these primary endpoints. Testing its sufficiency to augment effects of sodium deprivation (or to independently drive either of our primary endpoints) and testing the effects of potassium deprivation alone were subsidiary comparisons, which we investigated in smaller cohorts. Therefore, while our results do not support a major role for potassium, low statistical power prevents us from drawing strong conclusions as to whether elevated potassium may augment HSD2 neuronal activation and sodium appetite. Some of our results, such as the low‐level activation of HSD2 neurons in two potassium‐supplemented rats, hint that hyperkalemia may have an effect, as might be expected from increasing aldosterone, which promotes both HSD2 neuronal activity and sodium appetite (Gasparini et al., 2018; Resch et al., 2017).

Next, we used increased plasma protein as a proxy for reduced ECF volume. While the total plasma protein concentration of blood plasma is a reasonable proxy for changes in blood volume, it may underestimate the degree of hypovolemia (Kutscher, 1971), and we recognize that it is not an ideal indicator of volume status. Volume status was not a primary endpoint in this study, and in future work, more precise volume estimates could be obtained using classic dilution methods (Lee & Blaufox, 1985).

Finally, the relative unpalatability of potassium chloride may explain why potassium‐supplemented rats ingested saline when the 3% NaCl drinking tube was re‐introduced for the 2 h sodium appetite test. Favoring this interpretation, their total fluid intake during this test was similar to that of nonsupplemented controls. Nonetheless, it remains unclear whether this group (potassium‐supplemented; not sodium‐deprived) developed a mild sodium appetite or simply preferred to drink concentrated saline over a similarly unpalatable solution of concentrated potassium that had been available continuously for more than a week before this 2 h test.

### 4.2 Potassium is important for increasing aldosterone, but is not the key signal linking sodium deprivation to HSD2 neurons, and sodium appetite

Increasing pK via potassium supplementation greatly elevated plasma aldosterone, but did not increase HSD2 neuronal activation. Furthermore, saline consumption by hyperkalemic sodium‐deprived rats was no greater than hypokalemic sodium‐deprived rats. Saline intake in both these groups was slightly higher than that of rats that were simply sodium‐deprived, but the differences were small, and global analysis of the data (Figure 5D) revealed a broad spectrum of plasma potassium concentrations (3–8 mM) uncorrelated with HSD2 neuronal activation. Clearly, a rising potassium concentration is not the primary signal through which a sodium‐deficient diet promotes HSD2 neuronal activation.

Moreover, just as a hyperkalemic boost in aldosterone did not translate into elevated c‐Fos expression in the HSD2 neurons, the blunted aldosterone levels of potassium‐ and sodium‐deprived rats did not reduce HSD2 neuronal activation. Consistent with the idea that aldosterone helps promote HSD2 neuronal activation (and thereby sodium appetite), this hormone was elevated in all sodium‐restricted rats. Potassium restriction and supplementation exerted large influences in both directions—hypokalemic rats had half, and hyperkalemic rats more than double the aldosterone elevation produced by sodium deprivation alone. In fact, the potassium‐supplemented, hyperkalemic group had among the highest aldosterone levels reported in rodents, ranging 500–1000 ng/dl or higher. Values this high have been reported after dietary sodium deprivation in mice and in a human patient with an aldosterone‐secreting tumor (Okubo et al., 1997, 2016), but probably lie at the upper extreme for adrenal production of this hormone, which normally circulates at levels well below 100 ng/dl. The doubling of aldosterone in potassium‐supplemented, sodium‐deprived rats presumably reflects voltage‐dependent mechanisms that increase aldosterone released by cells in the zona glomerulosa (ZG) of the adrenal glands (Aguilera & Catt, 1986; Lotshaw, 2001), plus hypovolemic RAS activation, by which angiotensin II helps promote aldosterone synthesis and release by these same cells (Lotshaw, 2001; Spielman & Davis, 1974). In potassium‐deprived rats, the opposite likely occurred, with hypokalemia causing hyperpolarization of zona glomerulosa cell membranes and thereby blunting aldosterone release (Lotshaw, 2001) despite a presumably similar degree of hypovolemic RAS activation.

Despite their characteristic sensitivity to aldosterone, HSD2 neurons do not activate and inactivate based solely on the plasma aldosterone concentration. They are robustly activated by sodium deprivation in adrenalectomized rats, which have no circulating aldosterone (Geerling, Engeland, et al., 2006). Also, consuming saline reduces their activity despite persistent MR activation by an exogenous hormone (Geerling & Loewy, 2006). Remarkably, we show here that dietary sodium restriction similarly activates these aldosterone‐sensitive neurons across a wide range of aldosterone values, spanning 100–1000 ng/dl. Every sodium‐deprived rat had a large increase in c‐Fos activation of the HSD2 neurons, unaffected by supplementing or restricting potassium. Supplementing potassium alone caused hyperaldosteronism without significantly increasing HSD2 neuronal activation. Most remarkably, restricting dietary potassium greatly blunted the rise in plasma aldosterone caused by sodium deprivation without at all reducing c‐Fos immunoreactivity in HSD2 neurons. These rats, some with plasma aldosterone levels below 100 ng/dl, consumed just as much saline as rats with aldosterone levels several‐fold higher, confirming that aldosterone is not the primary stimulus for HSD2 neurons.

Eliminating pK as an important signal leaves open the question, what is the mechanistic link between sodium deprivation and sodium appetite? Restricting dietary sodium leads to an isotonic volume contraction, which reduces renal blood flow. This triggers renin release, resulting in the production of angiotensin II (AngII). AngII, in turn, activates thirst‐promoting neurons in the brain (Allen et al., 2017; Fitzsimons, 1998; Simpson et al., 1978), and it may synergize with aldosterone to promote sodium appetite (Epstein, 1982; Fluharty & Epstein, 1983; Sakai et al., 1986; Zhang et al., 1984). Infusing supraphysiologic amounts of AngII directly adjacent to the HSD2 neurons can promote saline consumption (Watson, 1986). HSD2 neurons express *Agtr1a*, which encodes the receptor for AngII, and they activate in response to exogenous AngII (Gasparini et al., 2019; Resch et al., 2017). AngII is widely believed to promote sodium appetite (Daniels & Fluharty, 2004; Fitzsimons, 1998; Johnson & Thunhorst, 1997; Lowell, 2019; Weisinger et al., 1996), but whether or not it is necessary remains unclear. In addition to hormones like aldosterone and AngII, synaptic input may play an important role in activating HSD2 neurons to promote sodium appetite. Vagal mechanoreceptors continuously monitor arterial pressure (and possibly cardiac preload) and deliver this information as synaptic input to the NTS. The HSD2 neurons receive very little of this input directly (Resch et al., 2017; Shin et al., 2009), but local interneurons may transform this information to activate the HSD2 neurons when blood volume dips (Sequeira et al., 2006). It is likely that a combination of synaptic and humoral input signals sculpts HSD2 neuronal activity, and thereby control sodium appetite, and this remains an important topic for future investigation.

## 5 CONCLUSIONS

Dietary sodium restriction causes sodium appetite in rats independently of potassium levels in the blood. Potassium potently influences aldosterone, but increasing or reducing pK and aldosterone does not significantly alter sodium appetite or HSD2 neuron activation in the sodium‐deficient state.

## CONFLICT OF INTERESTS

None of the authors have any conflict of interest related to this work.

## AUTHORS’ CONTRIBUTIONS

JCG and ADL planned the experiments. JCG performed the experiments, collected data, and conducted initial data analysis in ADL’s laboratory. FSF organized data and performed further analyses with JCG in JCG’s laboratory. FSF and JCG drafted and edited the manuscript and figures. All authors (except ADL) edited and approved the final version of this manuscript.

## Supporting information



Figure S1

Figure S2

## Data Availability

The data that support the findings of this study are available from the corresponding author upon reasonable request.
