# Supplementary figures and images for: Despite increasing aldosterone, elevated potassium is not necessary for activating aldosterone‐sensitive HSD2 neurons or sodium appetite
Source: Physiol Rep. 2021 Jan 19;9(2):e14714. doi: 10.14814/phy2.14714 (PMC7814482; doi:10.14814/phy2.14714)

A

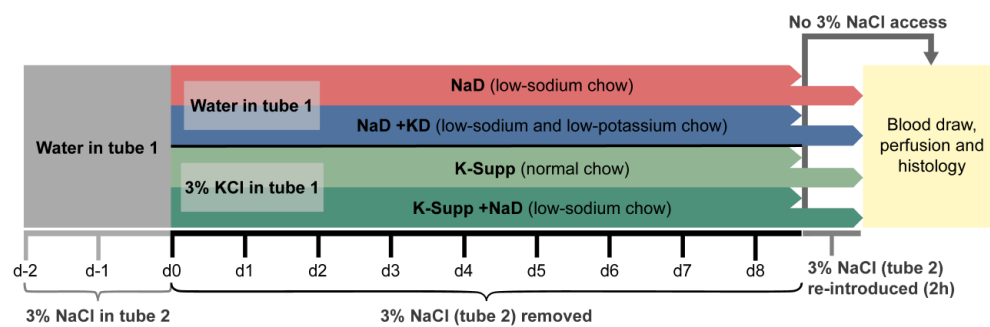

B

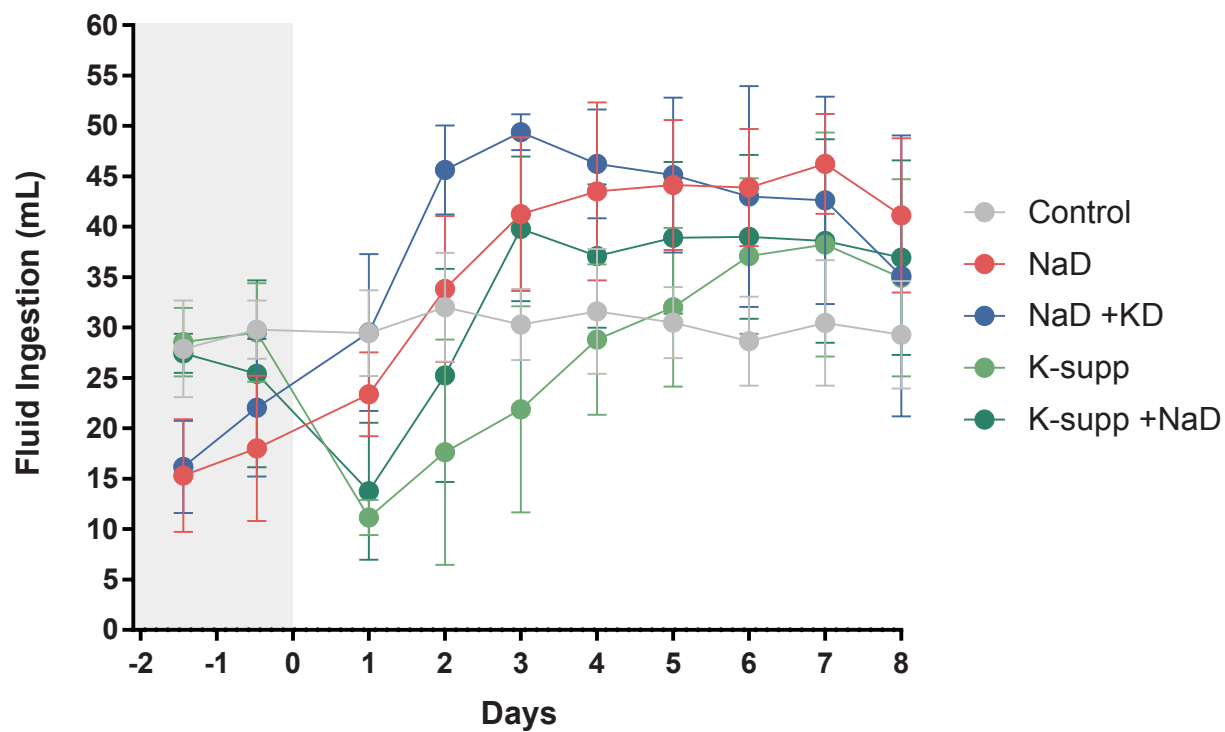

C

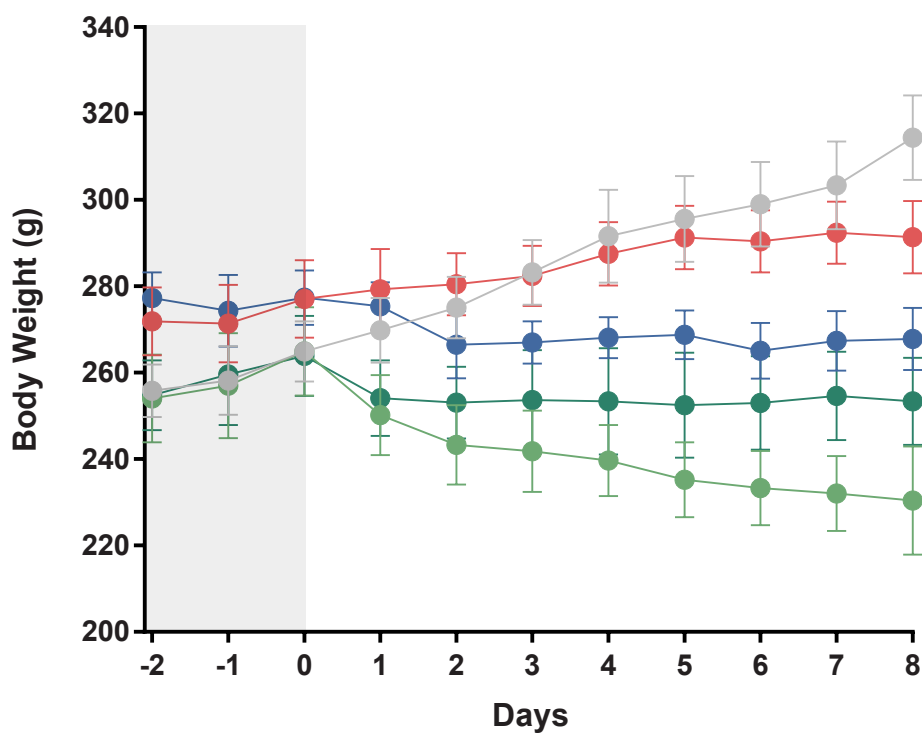

Supplement: Supplementary file 1 — Figure S1 [file PHY2-9-e14714-s001.pdf]

A

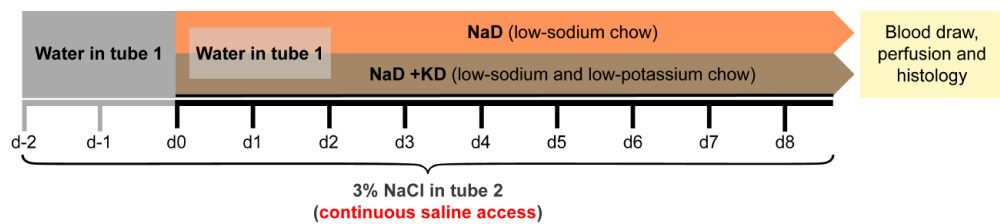

B

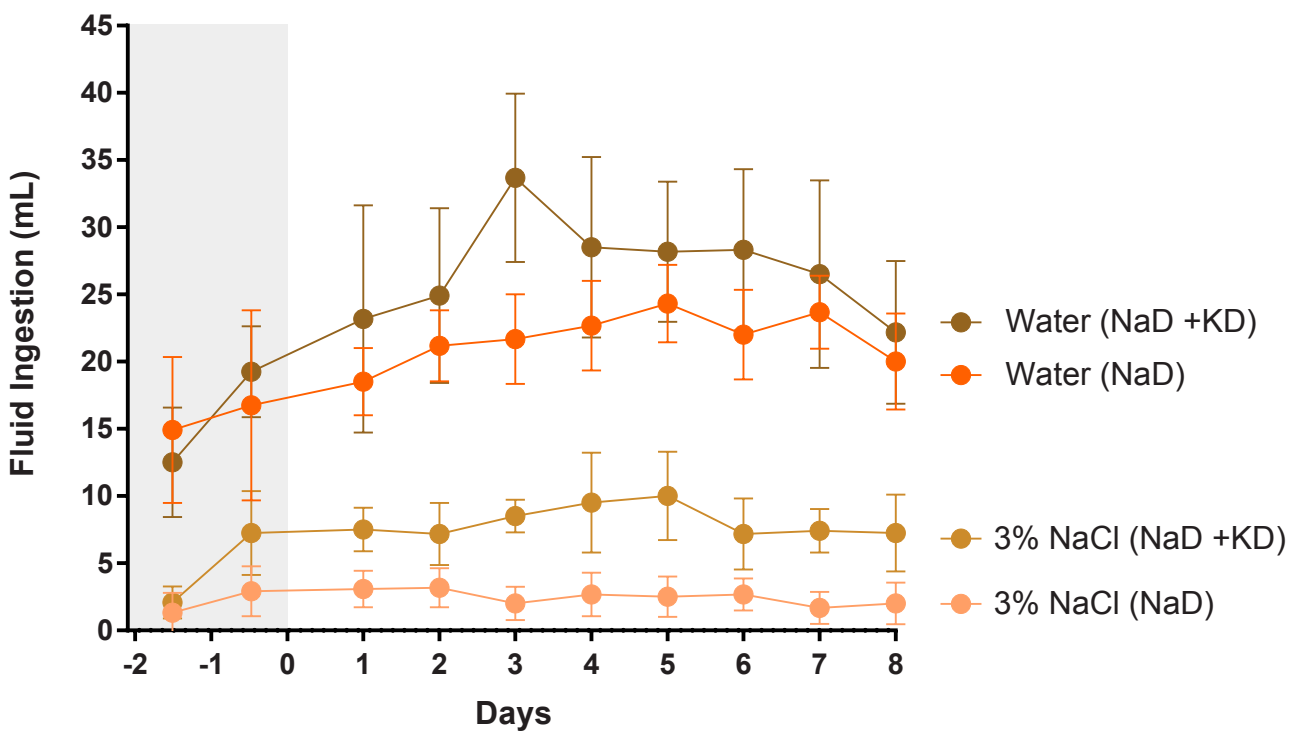

C

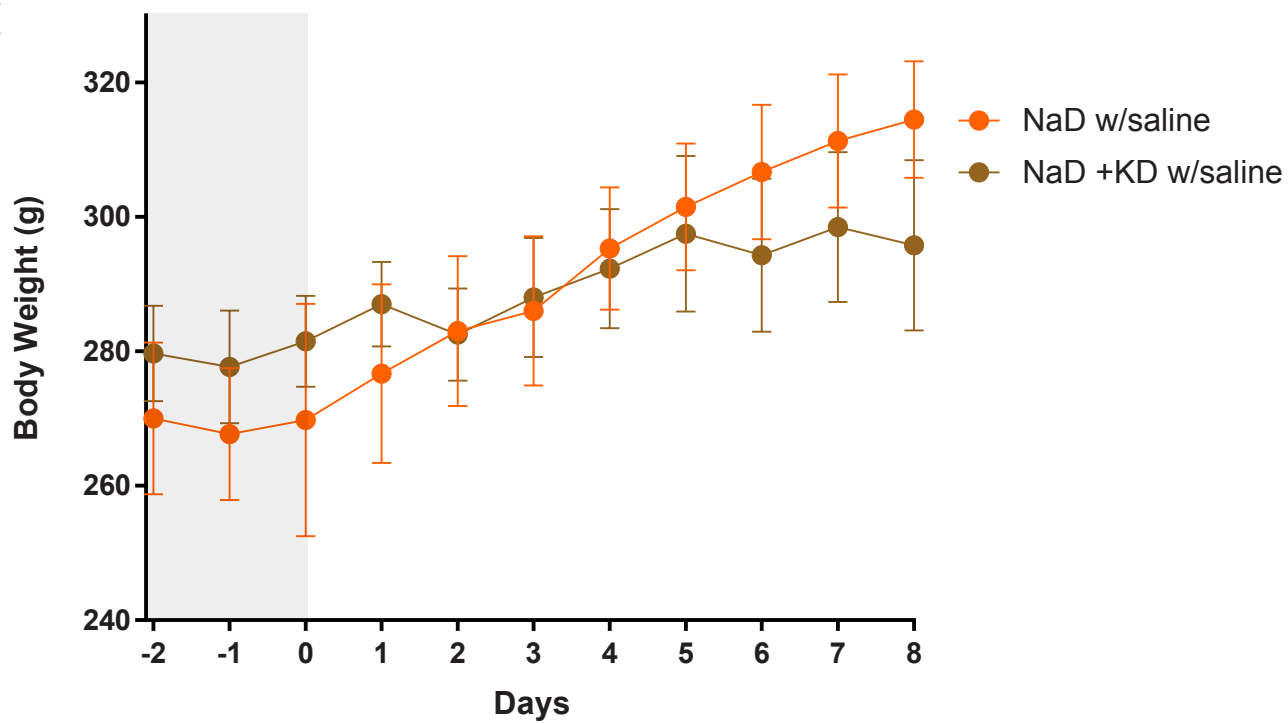

Supplement: Supplementary file 2 — Figure S2 [file PHY2-9-e14714-s002.pdf]
